# High viral abundance as a consequence of low viral decay in the Baltic Sea redoxcline

**DOI:** 10.1371/journal.pone.0178467

**Published:** 2017-06-08

**Authors:** Nicole Köstner, Lisa Scharnreitner, Klaus Jürgens, Matthias Labrenz, Gerhard J. Herndl, Christian Winter

**Affiliations:** 1 Department of Limnology and Bio-Oceanography, University of Vienna, Vienna, Austria; 2 Department of Biological Oceanography, Leibniz Institute for Baltic Sea Research (IOW), Rostock-Warnemünde, Germany; 3 Department of Marine Microbiology and Biogeochemistry, Royal Netherlands Institute for Sea Research, Utrecht University, AB Den Burg, The Netherlands; Universidad Miguel Hernandez de Elche, SPAIN

## Abstract

Throughout the Baltic Sea redoxcline, virus production and the frequency of lytically-infected prokaryotic cells were estimated from parallel incubations of undiluted seawater and seawater that contained prokaryotes with substantially reduced numbers of viruses (virus dilution approach), effectively preventing viral reinfection during the incubation period. Undiluted seawater incubations resulted in much higher estimates of virus production (6–35×10^4^ mL^-1^ h^-1^) and the frequency of infected cells (5–84%) than the virus dilution approach (virus production: 1–3×10^4^ mL^-1^ h^-1^; frequency of infected cells: 1–11%). Viral production and the frequency of infected cells from both approaches, however, cannot be directly compared, as data obtained from undiluted incubations were biased by viral reinfection and other uncontrollable processes during the incubation period. High *in situ* viral abundance (1–2×10^7^ mL^-1^) together with low virus production rates based on the virus dilution approach resulted in some of the longest viral turnover times (24–84 d) ever reported for the epipelagial. Throughout a wide range of environmental conditions, viral turnover time and burst size were negatively correlated. Given that viral decay estimated in ultra-filtered water was below the detection limit and the burst size was low (1–17), we conclude that prokaryotic viruses in the Baltic Sea redoxcline are investing most of their resources into stress defense (strong capsids) rather than proliferation (high burst size). In summary, the Baltic Sea redoxcline constitutes an environment where low virus production is found in combination with low viral decay, resulting in high viral abundance.

## Introduction

The Baltic Sea is among the largest brackish water systems on Earth. The discharge of major rivers (e.g., Neva, Vistula) leads to the establishment of a stable halocline between less saline water at the surface and the deeper more saline water originating from the North Sea. The absence of mixing leads to oxygen depletion in deeper water as a consequence of heterotrophic degradation of organic material. Under oxygen-free conditions, prokaryotes (here used to denote members of the phylogenetic domains *Bacteria* and *Archaea*; no phylogenetic relationship is implied) use trace metals, nitrate or sulfate as alternate electron acceptors [1]. The reduction of sulfate results in high concentrations of hydrogen sulfide (H_2_S) in deeper waters. The pelagic redoxcline is characterized by a steep redox gradient and is found between the well-oxygenated surface and the deeper sulfidic water. The water column of the Baltic Sea can be divided into four depth zones based on the concentration of O_2_ and H_2_S [2]. At the surface, the oxic zone is characterized by O_2_ in excess of 30 μM. The suboxic zone contains less than 30 μM O_2_, yet H_2_S is not detectable. In the transition zone, low O_2_ and H_2_S are found concurrently, followed by the anoxic zone with high H_2_S and no detectable O_2_. Episodic mixing between fresher surface and saltier deep waters may occur due to salt water inflows from the North Sea, leading to saline oceanic water filling large areas of the Baltic Sea. The last two reported major inflow events of North Sea water into the Baltic Sea occurred in the years 2003 [3] and 2014 [4], resulting in a complete ventilation of the water column, even of its deep basins.

Prokaryotic communities of the Baltic Sea redoxcline are phylogenetically similar to prokaryotes found in other oxygen minimum zones [5–7]. In general, the two principal prokaryotic mortality factors are protistan grazing and viral lysis (e.g., [8]). For the Baltic Sea, Anderson and colleagues [2] showed that protistan grazing is relevant in oxygenated waters, where 50–100% of the prokaryotic standing stock is grazed per day, but is negligible in anoxic waters. Virus proliferation depends on host physiology, counteracted by virus decay, either through loss of infectivity or virus particle destruction. A variety of externally-driven virus decay mechanisms has been identified such as ultraviolet radiation [9], heat-sensitive substances (e.g., extracellular enzymes, [9,10]), adsorption to particles [11], changing temperatures [12,13], and protistan grazing on viruses (virivory, [14–16]). Also, internal pressure caused by packaging the viral genome into relatively small capsids [17] may cause viral decay of viruses infecting prokaryotes that is intrinsically defined and does not depend on external factors [18]. Furthermore, experimental data show that viruses face the same problem of allocating limited resources either into proliferation (high burst size; the number of viruses released per prokaryotic cell) or defense mechanisms (high virus capsid strength counteracting internal pressure, [18]) as do living organisms.

Viral lysis of prokaryotes in the redoxcline of the Baltic Sea was studied twice (in September 1998 [19] and in September 2009 [2]), using different methods and leading to contradictory results. Weinbauer et al. [19] applied transmission electron microscopy (TEM) to *in situ* samples at two stations in the Central Baltic Sea to determine the frequency of visibly infected prokaryotic cells and the burst size. Furthermore they related the frequency of visibly infected prokaryotic cells to the frequency of lytically infected prokaryotic cells (FIC) using a conversion factor derived from virus dilution incubations and TEM observations at the same study site [20]. Furthermore, Weinbauer et al. [19] estimated virus production (VP) by multiplying prokaryotic cell production with FIC/100 and the burst size. To distinguish between FIC and lysogenically infected prokaryotic cells (FLC) they compared the temporal development of viral abundance in parallel incubations of 10 μm-filtered water with and without mitomycin C [21]. Weinbauer et al. [19] reported increasing FIC from the suboxic (9–13%) to the anoxic zone (17–25%) while FLC was highest in the suboxic zone (16–44%) and decreased towards the anoxic zone (4–9%). In contrast, Anderson and colleagues [2] used the virus dilution approach [22] to estimate FIC and FLC based on changes in viral abundance over an incubation period of up to 30 h [23]. The rationale behind this approach is to prevent new viral infections during the incubations by removing most of the viruses by differential filtration, because virus-host encounter rates are abundance-dependent [24]. Consequently, increasing viral abundance during the incubation is a result of infections from before the sample was taken. In such samples, complex communities of prokaryotic taxa with a certain fraction infected by an even more diverse virus community are studied. Many of these host cells might be in varying stages of viral infection (from attachment of the infecting virus to just before lysis of the host cell), some host cells might even be infected by more than one virus taxon [25], and many viruses might differ in latency period, burst size, and intrinsic decay rates [18]. This diversity may result in various discrete lysis events, detectable as maxima in viral abundance, especially at short sampling intervals. Thus, Anderson et al. [2] calculated FIC based on virus dilution incubations by taking the observed maxima into account as previously published [23]. This is distinctly different to the approach of Weinbauer et al. [19], who did not remove viruses allowing for new infections during the incubation. Anderson and colleagues [2] found FIC to decrease from the suboxic (6–19%) to the anoxic zone (2–3%) while FLC could not be detected.

These discrepancies in the magnitude and relationship of FIC and FLC with depth throughout the redoxcline point towards a lack of understanding virus-mediated prokaryotic mortality in the Baltic Sea redoxcline, aggravated by differences in sampling date, study design, and data treatment [2,19]. Additionally, the low virus-mediated prokaryotic mortality obtained from the virus dilution approach [2] appears to be incompatible to the high viral abundance generally found in the Baltic Sea [26,27]. Here we used two approaches, undiluted and virus dilution incubations, under identical conditions and from the same water samples throughout the Baltic Sea redoxcline. The temporal developments of prokaryotic and viral abundance during the 40 h incubation period were used to determine rates of prokaryotic growth and mortality as well as viral production and decline. Special attention was given to the prevalent, low O_2_ or the complete lack thereof. The aim of the study was to identify strategies of the viral community explaining the discrepancy between high *in situ* viral abundance and low virus-mediated prokaryotic mortality [2]. We hypothesized that overall low virus-mediated prokaryotic mortality increases from the oxic to the anoxic zone as protistan grazing is known to decrease with decreasing oxygen concentration [2,28]. Additionally, we hypothesized that VP and FIC are higher in undiluted than in virus dilution incubations.

## Materials and methods

### Ethics statement

**Fig 1:**
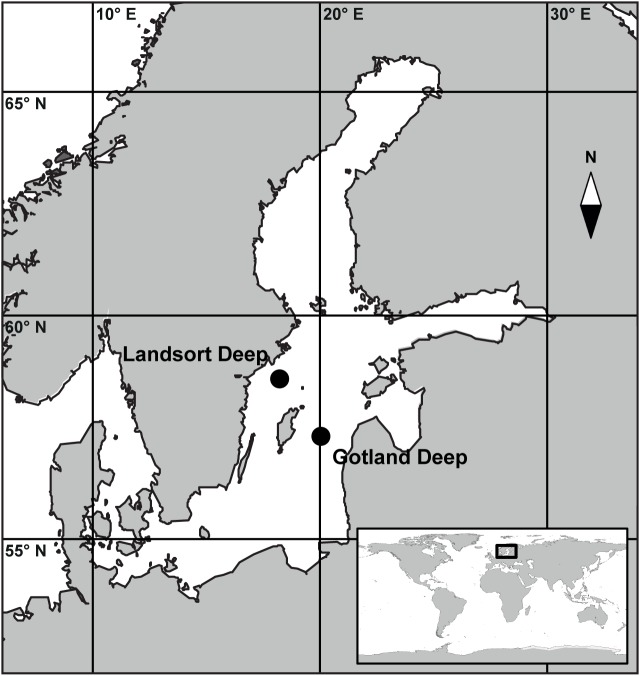
Map of the Baltic Sea. The map shows the location of the sampling stations within the Central Baltic Sea. The main map is marked by the black square within the global map in the inset. Both maps were generated with the software package Ocean Data View [30].

No specific permissions were required for the two sampling locations (Fig 1) and activities conducted in this study. No endangered or protected species were involved.

### Sampling and physicochemical parameters

Sampling was conducted once at Gotland Deep (57°19' N, 20°03' E, bottom depth: 248 m) and twice at Landsort Deep (58° 35' N, 18° 14' E, bottom depth: 460 m) in June 2012 (Fig 1). Salinity, temperature, turbidity, and O_2_ were recorded by sensors mounted on the sampling rosette (Seabird SBE 911plus, Sea-Bird Electronics Inc., Bellevue, WA, USA); data were used to determine the depth of the suboxic, transition, and anoxic zones. Samples (2 L) were withdrawn from the water sampler into polycarbonate bottles allowing the containers to overflow with at least 0.5 L to avoid oxygen contamination. The concentration of NO_2_, NO_3_, NH_4_, PO_4_, O_2_, and H_2_S were determined on board using standard protocols [29]. Generally, the sampling depth was determined based on downward profiles of O_2_ measured by the oxygen sensor on the sampling rosette, with the goal to cover the suboxic, transition, and anoxic zone at each location. However, subsequent on board determination of O_2_ using the Winkler method and H_2_S using a spectrophotometric analysis method [29] occasionally led to changes in assigning water samples to depth zones (Table 1).

**Table 1:** Physicochemical parameters at Gotland Deep and Landsort Deep.

Sampling location	Depth zone	Depth	Temp	Sal	Turb	PO_4_		NO_2_		NO_3_		NH_4_		O_2_		H_2_S	
		(m)	(°C)		(ntu)	(μM)	(μg/L)	(μM)	(μg/L)	(μM)	(μg/L)	(μM)	(μg/L)	(μM)	(μg/L)	(μM)	(μg/L)
Gotland Deep	Oxic Zone	80	4.77	9.32	0.06	2.2	208.8	0.3	13.1	1.0	62.6	1.0	17.9	41.4	1326.2	n.d.	n.d.
	Transition Zone	90	5.25	10.18	0.10	3.6	346.6	0.7	31.8	n.d.	n.d.	1.4	25.6	0.6	19.3	1.3	43.4
	Anoxic Zone	95	5.32	10.34	0.31	3.5	333.2	0.4	18.7	n.d.	n.d.	2.1	38.1	n.d.	n.d.	3.2	108.9
Landsort Deep 1	Suboxic Zone	75	5.21	9.57	0.18	2.9	279.6	0.4	16.8	3.6	220.6	0.4	7.2	13.5	430.7	n.d.	n.d.
	Transition Zone	85	5.42	9.95	0.09	3.5	328.4	0.2	10.3	n.d.	n.d.	3.1	56.8	1.3	40.3	0.2	6.7
	Anoxic Zone	95	5.49	10.07	0.51	3.5	328.4	0.1	4.7	n.d.	n.d.	4.2	76.6	n.d.	n.d.	4.6	156.3
Landsort Deep 2	Transition Zone	78	5.26	9.61	0.18	3.4	325.6	0.3	15.8	4.5	279.0	<0.1	<0.1	4.4	142.3	0.4	13.4
	Anoxic Zone 1	90	5.43	9.95	0.24	3.7	350.2	<0.1	1.9	<0.1	<0.1	3.4	60.7	n.d.	n.d.	0.3	9.4
	Anoxic Zone 2	100	5.50	10.10	0.43	3.4	323.3	<0.1	1.9	n.d.	n.d.	4.0	72.1	n.d.	n.d.	4.0	135.6

The table gives depth, temperature (Temp), salinity (Sal), turbidity (Turb; nephelometric turbidity units), PO_4_, NO_2_, NO_3_, NH_4_, O_2_, and H_2_S at Gotland Deep, Landsort Deep 1, and Landsort Deep 2. Depth zone classification was based on O_2_ and H_2_S. Values below the detection limit are indicated as not detectable (n.d.).

### Determination of prokaryotic and viral abundance

Samples for enumerating prokaryotes and viruses were fixed with glutaraldehyde (0.5% final concentration), flash-frozen in liquid nitrogen, and stored at -80°C. Enumeration of prokaryotes and viruses was performed according to previously published protocols [31,32]. Briefly, upon thawing, samples were diluted in an equal volume of Tris-EDTA buffer (TE: 10 mM Trishydroxymethyl-aminomethane, 1 mM ethylenediaminetetraacetic acid; SIGMA Aldrich, St. Louis, MO, USA; pH 8.0). Subsequently, double-stranded DNA found in the genomes of prokaryotes and viruses was stained with SYBR Green I (final concentration: 1:20 000 dilution of 10 000× commercial stock, Invitrogen, Life Technologies, Carlsbad, CA, USA) in the dark for 10 min. For prokaryotes, staining was performed at room temperature and for viruses at 80°C. Triplicates of each sample were counted on a BD FACSAria II flow cytometer (Becton Dickinson, Durham, NC, USA). In order to prevent coincidence on the flow cytometer when measuring the samples, particle event rates were kept below 1000 events s^-1^ by further diluting in Tris-EDTA buffer as needed. Gating was performed on cytograms of side scatter versus green fluorescence.

### Experimental setup and estimation of prokaryotic and viral performance

**Fig 2:**
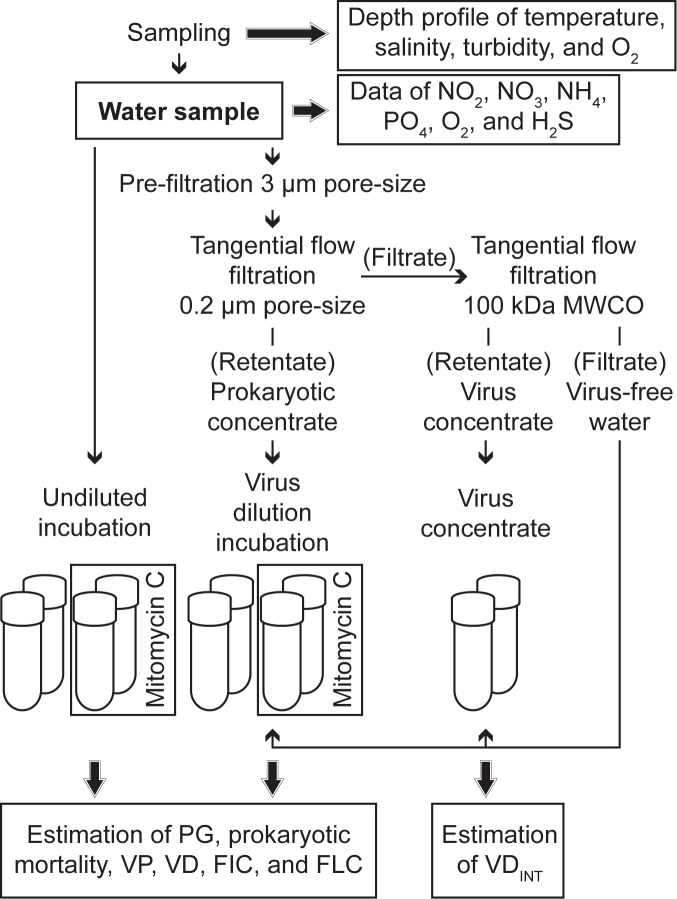
Experimental setup. The figure shows the experimental setup of undiluted and virus dilution incubations to determine rates of prokaryotic growth (PG), prokaryotic mortality, virus production (VP), viral decay (VD), and the frequency of lytically (FIC) and lysogenically infected prokaryotic cells (FLC). In parallel, intrinsic viral decay (VD_INT_) was estimated by incubating virus concentrate in virus-free water.

Experiments, each consisting of 5 treatments in duplicate, were performed from each sample (Fig 2). Sample handling (filtrations, setup, subsampling) was performed in an anaerobic chamber filled with nitrogen gas. Experiments were performed in 60 mL gas-tight glass vials (Cat. No. SU860003 and 27022; SIGMA Aldrich). For undiluted incubations, water (50 mL each) was directly dispensed into 4 glass vials. A subset of 2 vials was amended with mitomycin C (Cat. No. M0503_2MG, SIGMA Aldrich; 1 μg mL^-1^ final concentration; Fig 2) to induce lysogenic viruses. For the virus dilution approach [22], water (1.8 L) was filtered over 3 μm pore size filters (Cat. No. TSTP04700, 47 mm diameter; Merck Millipore, Darmstadt, Germany). Prokaryotes were concentrated by tangential flow ultra-filtration (Vivaflow 200, PES membrane, 0.2 μm pore size, Cat. No. VF20P7; Sartorius Stedim Biotech, Göttingen, Germany) until the retentate volume was below 100 mL. Finally, virus concentrate and virus-free water was obtained by tangential flow ultra-filtration (Vivaflow 200, PES membrane, molecular weight cut-off 100 kDa, Cat. No. VF20P4, Sartorius Stedim Biotech). Virus dilution incubations (total volume of 50 mL) consisted of 5 mL of prokaryotic concentrate inoculated into 45 mL of virus-free water with a subset of the incubations (2 out of 4 vials in total) amended with mitomycin C as for undiluted incubations (Fig 2). To determine intrinsic viral decay (excluding all external viral decay mechanisms; VD_INT_), 5 mL of virus concentrate were inoculated into 45 mL of virus-free water (total volume of 50 mL), both obtained by the second filtration step of the original sample. All incubations were performed in the dark at 4°C for 40 h. Every 5 h, a 1.8-mL subsample was collected from each incubation to determine prokaryotic and viral abundance. At the end of the incubation period, samples to determine the relative abundance of *Bacteria*, *Crenarchaeota*, and *Euryarchaeota* were taken and processed as described below.

Changes in prokaryotic and viral abundance over the incubation period were used to estimate rates of prokaryotic growth (PG), prokaryotic mortality, VP, viral decay (VD), and FIC from each duplicate of undiluted and virus dilution incubations without mitomycin C (S1 Table, S1 Fig). PG and VP were calculated from positive slopes between local minima and maxima of prokaryotic and viral abundance, respectively (S1 Table equations 1 and 5, S1 Fig; [23]). Accordingly, prokaryotic mortality and VD were calculated from negative slopes between local maxima and minima of prokaryotic and viral abundance, respectively (S1 Table equations 2 and 6, S1 Fig). It is well known that recover efficiencies of prokaryotes (and viruses) from tangential flow filtration devices vary between runs of the same sample as well as among samples [33]. Thus, all rates estimated from the virus dilution approach were corrected for differences between *in situ* prokaryotic abundance and prokaryotic abundance at the start of the experiments. For comparing both approaches, we calculated PG corrected for prokaryotic mortality and VP corrected for VD (S1 Table equations 3 and 7) to estimate the average rate of change in prokaryotic and viral abundance over the incubation period, taking into account also loss rates due to mortality and decay, respectively. FIC was calculated based on local minima and maxima of viral abundance and prokaryotic abundance at the start of the experiments (S1 Table equation 9, S1 Fig; [23]). A constant burst size of 28 viruses per lysed cell was used in these calculations as determined by Weinbauer and colleagues [19] using TEM for the suboxic zone of the Baltic Sea. FLC was calculated as the difference in FIC between mitomycin C-treated and untreated incubations, provided the ranges were not overlapping between both treatments and the average value of the mitomycin C-treated incubations was higher than in the uninduced incubations. Specifically, data are calculated from the duplicate incubations; average values and ranges are computed and compared between uninduced and mitomycin C-treated incubations. Prokaryotic and viral turnover times were calculated by dividing *in situ* abundances through PG and VP, respectively (S1 Table equations 4 and 8). VD_INT_ was calculated from parallel viral decay incubations (incubations of virus concentrate inoculated into virus-free water) as the slope of a linear least-squares regression of the natural logarithm of viral abundance over incubation time [23,34]. Generally, data from the incubations are presented as the average and range of duplicate incubations.

### Determination of the relative abundance of *Bacteria*, *Crenarchaeota*, and *Euryarchaeota*

The relative abundance of *Bacteria*, *Crenarchaeota*, and *Euryarchaeota* was determined by catalyzed reporter deposition-fluorescence *in situ* hybridization (CARD-FISH, [35]). Samples (10–35 mL) were fixed with formaldehyde (2% final concentration) and stored in the dark at 4°C for 24 h. Subsequently, prokaryotic cells were collected onto white polycarbonate filters (GTTP, 25 mm diameter, 0.22 μm pore size, Cat. No. GTTP02500, Merck Millipore); the filters were air-dried and stored in 2 mL cryovials at -80°C until further analysis. To enumerate *Bacteria*, we used an equimolar mix of probes EUB338 (GCTGCCTCCCGTAGGAGT; [36]), EUB338-II (GCAGCCACCCGTAGGTGT; [37]), and EUB338-III (GCTGCCACCCGTAGGTGT; [37]). For the detection of *Crenarchaeota*, an equimolar mix of probes CREN537 (TGACCACTTGAGGTGCTG; [35]) and GI554 (TTAGGCCCAATAATCMTCCT; [38]) was used. At Gotland Deep, we used the probe EURY806 (CACAGCGTTTACACCTAG; [35]) to detect *Euryarchaeota*. Hybridization, signal amplification, and mounting of filter slices onto slides followed Teira and colleagues [35]. The number of probe-positive cells and 4’,6’-diamidino-2-phenylindole (DAPI)-stained cells was determined in 20 fields of view on an Axio Imager M2 epifluorescence microscope (Zeiss, Jena, Germany) equipped with filter sets for DAPI (Cat. No. 48804999010001, Zeiss) and Alexa488 (Cat. No. 000000 1114 459, Zeiss). For *in situ* samples, the results represent data from a single sample, whereas experimental data were obtained from duplicate incubations and are given as average and range. Data are expressed as percentage of probe-positive cells relative to DAPI-stained cells.

### Statistical analysis

The Spearman rank correlation coefficient (*r*) was used to identify statistically relevant correlations between parameters, e.g., among physicochemical (depth, temperature, salinity, turbidity, NO_2_, NH_4_, PO_4_, O_2_, H_2_S) and biological parameters (prokaryotic and viral abundance, virus-to-prokaryote ratio). NO_3_ was excluded from statistical analyses, as it was not detectable in most samples. Redundancy analysis and subsequent variation partitioning was performed to test whether and to what extent the variations in a set of parameters (e.g., VP and FIC) could be explained by a set of explanatory parameters (e.g., NO_2_, NH_4_, PO_4_) given a set of conditional parameters (e.g., temperature and salinity defining water masses). For redundancy analysis, data were centered on their means and the significance of the results was tested with a permutation test (10 000 random permutations, re-sampling with replacement). A Mann-Whitney *U*-test was used to test for significant differences between two parameters (e.g., VP estimated by undiluted and virus dilution incubations). A Kruskal-Wallis test followed by a Mann-Whitney *U*-test as the non-parametric equivalent of a post-hoc test was used to test for significant differences in a parameter obtained in three different instances (e.g., the relative abundance of *Bacteria* among *in situ* samples and at the end of undiluted and virus dilution incubations). Values below the detection limit for specific parameters were assumed to be zero. The results of statistical analyses were assumed to be significant at *p*-values ≤ 0.05; in case of a comparison between single experiments, a relevant difference was assumed when the ranges of duplicate incubations were not overlapping. Statistical analyses were performed in SPSS (version 22) and R [39] using the packages “car” [40] and “vegan” [41].

## Results

### Physicochemical parameters

The water column at both study sites was stratified with a halocline at 70 m depth at Gotland Deep and at 53–56 m depth at Landsort Deep 1 and 2. First appearance of H_2_S was at 90 m depth at Gotland Deep and at 78 m depth at Landsort Deep 1 and 2. Temperature (4.8–5.5°C), salinity (9.32–10.34), and turbidity (0.06–0.51 nephelometric turbidity units) were positively correlated to depth (Spearman rank correlation coefficient: temperature: *r* = 0.765, *p* = 0.016, *N* = 9; salinity: *r* = 0.814, *p* = 0.008, *N* = 9; turbidity: *r* = 0.681, *p* = 0.44, *N* = 9; Table 1). NH_4_ (<0.1–4.3 μM) and H_2_S (0.2–4.6 μM) increased while O_2_ (0.6–41.4 μM) decreased with depth (Spearman rank correlation coefficient: NH_4_: *r* = 0.832, *p* = 0.005, *N* = 9; H_2_S: *r* = 0.844, *p* = 0.004, *N* = 9; O_2_: *r* = -0.891, *p* = 0.001, *N* = 9; Table 1) and temperature (Spearman rank correlation coefficient: NH_4_: *r* = 0.867, *p* = 0.002, *N* = 9; H_2_S: *r* = 0.711, *p* = 0.032, *N* = 9; O_2_: *r* = -0.844, *p* = 0.004, *N* = 9; Table 1). PO_4_ (2.2–3.7 μM) and NO_2_ (<0.1–0.7μM) were not correlated to depth. NO_3_ ranged from <0.1–4.5 μM.

### Prokaryotic and viral abundance

At Gotland Deep, prokaryotic abundance was substantially higher in the transition (9.0×10^5^ mL^-1^) and anoxic zone (7.3×10^5^ mL^-1^) as compared to the oxic zone (2.1×10^5^ mL^-1^; Table 2). At Landsort Deep 1, prokaryotic abundance was lowest in the suboxic zone (5.4×10^5^ mL^-1^), and increased towards the transition (9.8×10^5^ mL^-1^) and anoxic zone (8.9×10^5^ mL^-1^; Table 2). At Landsort Deep 2, prokaryotic abundance increased substantially from the transition zone (5.4×10^5^ mL^-1^) to the anoxic zone (8.8–9.3×10^5^ mL^-1^). Viral abundance did not differ among depth zones at any of the sampling stations and ranged from 1.5–1.9×10^7^ mL^-1^ at Gotland Deep, from 1.4–1.6×10^7^ mL^-1^ at Landsort Deep 1, and from 1.2–1.5×10^7^ mL^-1^ at Landsort Deep 2 (Table 2). Overall, the virus-to-prokaryote ratio varied from 16–90 and was highest in the oxic zone at Gotland Deep (Table 2). Prokaryotic abundance correlated positively with temperature (Spearman rank correlation coefficient: *r* = 0.667, *p* = 0.050, *N* = 9) and NH_4_ (Spearman rank correlation coefficient: *r* = 0.683, *p* = 0.042, *N* = 9), while viral abundance did not correlate with any of the recorded parameters. The virus-to-prokaryote ratio was negatively correlated to temperature (Spearman rank correlation coefficient: *r* = -0.833, *p* = 0.005, *N* = 9) and NH_4_ (Spearman rank correlation coefficient: *r* = -0.767, *p* = 0.016, *N* = 9).

**Table 2:** Abundance and turnover times of prokaryotes and viruses.

Sampling location	Depth zone	Prokaryotes		Viruses		Virus-to-prokaryote ratio	Prokaryotic turnover time		Viral turnover time	
		*Avg*	*SD*	*Avg*	*SD*		Undiluted	Virus dilution	Undiluted	Virus dilution
Gotland Deep	Oxic Zone	2.1	<0.1	1.9	0.3	90	2.0	3.6	6.5	42.3
	Transition Zone	9.0	0.2	1.7	1.2	18	5.6	7.1	4.7	53.6
	Anoxic Zone	7.3	0.3	1.5	0.4	20	4.3	7.7	11.8	69.8
Landsort Deep 1	Suboxic Zone	5.4	0.1	1.4	0.3	25	3.8	14.7	3.3	84.3
	Transition Zone	9.8	0.3	1.6	0.2	16	6.2	8.1	2.0	27.9
	Anoxic Zone	8.9	0.2	1.6	0.4	18	4.3	6.2	3.5	24.1
Landsort Deep 2	Transition Zone	5.4	0.1	1.2	0.5	22	4.6	10.6	2.4	25.0
	Anoxic Zone 1	8.8	0.4	1.4	0.6	16	13.0	15.6	6.1	28.4
	Anoxic Zone 2	9.3	0.3	1.5	0.9	16	1.9	7.0	5.4	37.2

Abundances (prokaryotes: N×10^5^ ml^-1^, viruses: N×10^7^ ml^-1^) are given as average (*Avg*) and standard deviation (*SD*). Prokaryotic and viral turnover times (S1 Table, equations 4 and 8; d) for undiluted and virus dilution incubations are given as the average of duplicate incubations at Gotland Deep, Landsort Deep 1, and Landsort Deep 2.

### Relative abundance of *Bacteria*, *Crenarchaeota*, and E*uryarchaeota*

*In situ* relative abundance of *Bacteria* ranged from 33–115% of DAPI-positive cells (S2A, S2C and S2E Fig). The relative abundance of *Crenarchaeota* did not show a clear trend with depth and varied from 8–35% (S2B, S2D and S2F Fig). *Euryarchaeota* were not detectable at Gotland Deep. At the end of the incubation period, relative abundance of *Bacteria* ranged from 39–95% in undiluted and from 47–81% in virus dilution incubations (S2A, S2C and S2E Fig), whereas the relative abundance of *Crenarchaeota* ranged from 2–33% in undiluted and from 8–41% in virus dilution incubations (S2B, S2D and S2F Fig). When comparing specific depth zones, *in situ* relative abundance of *Bacteria* was similar to corresponding samples from undiluted and virus dilution incubations (S2 Table). Taking all data into account, the relative abundance of *Bacteria* was significantly higher in undiluted incubations as compared to *in situ* samples (Mann-Whitney test: *U* = 2.2, *p* = 0.0268) and virus dilution incubations (Mann-Whitney test: *U* = 2.1, *p* = 0.0371), but was similar in *in situ* samples and virus dilution incubations (Mann-Whitney test: *U* = 0.9, *p* = 0.4033). Based on depth zone-specific as well as all data comparisons, the relative abundance of *Crenarchaeota* did not differ among *in situ* samples and any of the incubations (S2 Table).

### Virus-induced mortality of prokaryotes and VD_INT_

**Fig 3:**
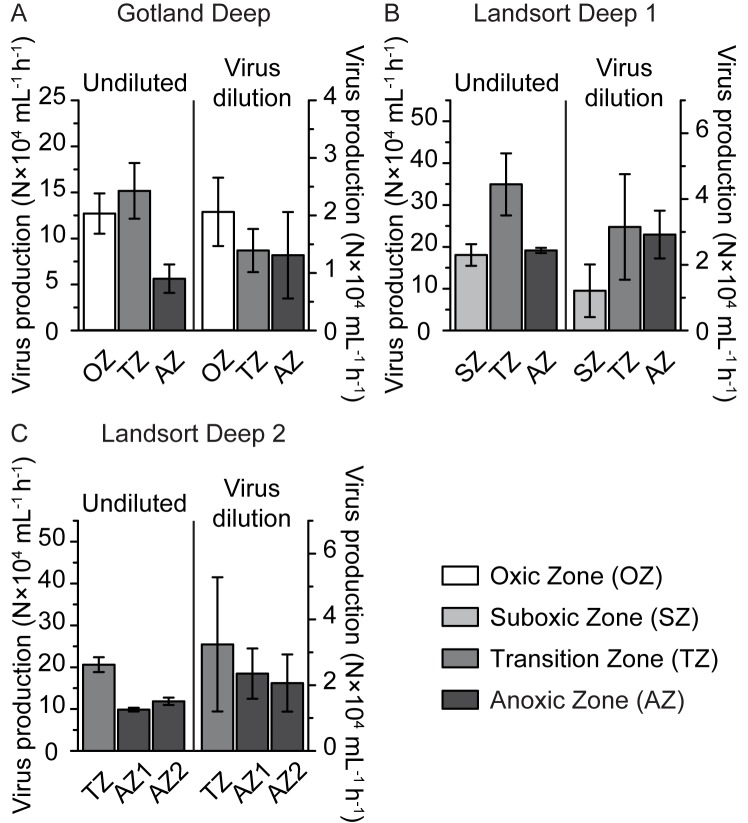
Virus production (VP) in undiluted and virus dilution incubations. The figure shows VP at Gotland Deep (A) and Landsort Deep 1 (B) and 2 (C). Rates are given as average values of duplicate incubations in the oxic (OZ), suboxic (SZ), transition (TZ), and anoxic zone (AZ). Error bars represent the range of duplicate incubations.

**Fig 4:**
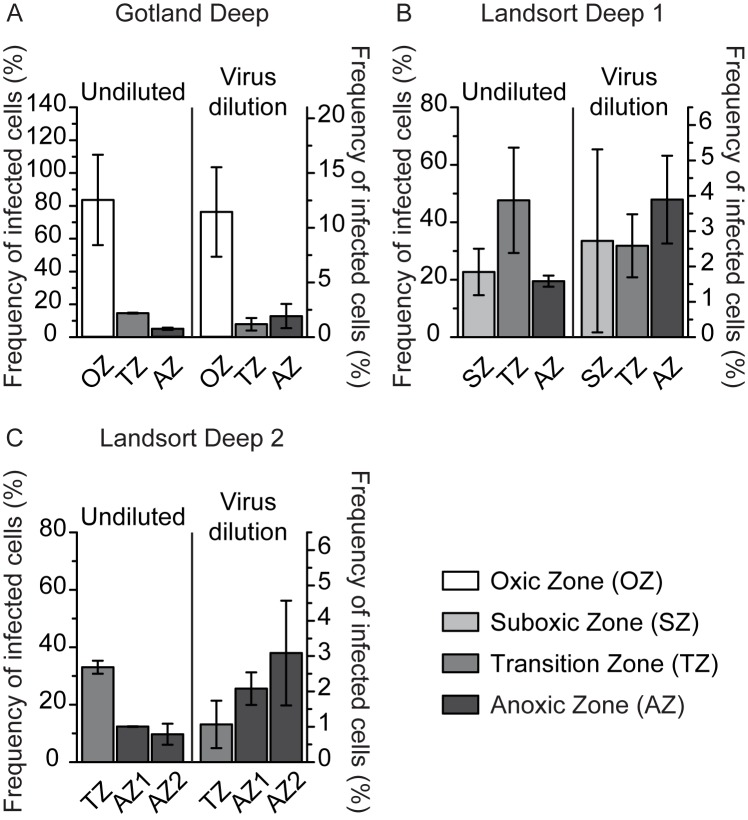
Frequency of lytically infected cells (FIC) in undiluted and virus dilution incubations. The figure shows FIC at Goland Deep (A) and Landsort Deep 1 (B) and 2 (C). FIC is given as the average of duplicate incubations in the oxic (OZ), suboxic (SZ), transition (TZ), and anoxic zone (AZ). Error bars represent the range of duplicate incubations.

In undiluted incubations, VP ranged from 5.6–34.9×10^4^ mL^-1^ h^-1^ (Fig 3) and FIC from 5.1–83.6% (Fig 4); VP and FIC were substantially higher in the transition zone as compared to the anoxic zone at all sampling stations (Figs 3 and 4). VP estimated in virus dilution incubations did not vary substantially among depth zones at any of the study sites, ranging from 1.3–2.1×10^4^ mL^-1^ h^-1^ at Gotland Deep and 1.2–3.2×10^4^ mL^-1^ h^-1^ at Landsort Deep 1 and 2 (Fig 3); highest FIC (11.4%) was found in the oxic zone at Gotland Deep, while FIC did not vary substantially among any other depth zones, ranging from 1.1–3.9% (Fig 4). A meaningful comparison of VP and FIC among sampling zones was only possible for the transition and anoxic zones, because the oxic and suboxic zone were only sampled once (Table 1, Figs 3 and 4). VP and FIC estimated from undiluted incubations were substantially lower in the transition and anoxic zones at Gotland Deep compared to Landsort Deep 1 and 2 (Figs 3 and 4). However, data from the virus dilution approach for the transition and anoxic zones did not differ among the sampling stations (Figs 3 and 4). In undiluted incubations, FLC could only be calculated once, amounting to 47% of *in situ* prokaryotic abundance in the transition zone at Landsort Deep 2, but could never be calculated in virus dilution incubations.

In incubations used to estimate VD_INT_, initial viral abundance ranged from 78–357% of *in situ* abundance. VD_INT_ was below the detection limit at all sampling stations and depth zones as estimates were statistically insignificant and poorly supported by their coefficients of determination in linear least-squares regression analyses (range of all incubations: *p* = 0.0859–0.7288; *R*^*2*^ = 0.02–0.37).

### Comparing data obtained from undiluted and virus dilution incubations

Overall, the temporal development of prokaryotic and viral abundance in undiluted and virus dilution incubations was never linear (S1 File, S1 Fig). In virus dilution incubations, prokaryotic and viral abundance at the beginning of the experiments ranged from 72–251% (average 132%) and from 10–53% (average 30%) of *in situ* abundance, respectively. Overall, the virus-to-prokaryote ratio in virus dilution incubations ranged from 5–68% of *in situ* levels (Table 2) throughout the incubations.

VP, VD, VP corrected for VD, and FIC estimated in undiluted incubations were significantly higher compared to virus dilution incubations (Mann-Whitney test: VP: *U* = 5.1, *p* < 0.0001; VD: *U* = 4.4, *p* < 0.0001; VP corrected for VD: *U* = 3.1, *p* = 0.0013; FIC: *U* = 4.7, *p* < 0.0001; S5 Table, Figs 3 and 4). Also, VP and FIC estimated by the two approaches were not correlated with each other (VP: Spearman rank correlation coefficient: *r* = 0.02, *p* = 0.9345, *N* = 18; FIC: Spearman rank correlation coefficient: *r* = 0.12, *p* = 0.6266, *N* = 18). Variation partitioning indicated that the variation in FIC and VP estimated by undiluted incubations could not be explained by a model containing *in situ* NO_2_, NH_4_, and PO_4_ as explanatory variables and temperature and salinity as conditional variables (S3 Table). However, the same model explained the variability of FIC and VP estimated by virus dilution incubations well (variation explained: 93%; S3 Table). In particular, NO_2_, NH_4_, and PO_4_, corrected for the influence of temperature and salinity, explained 41% of the variation in FIC and VP (S3 Table). In contrast, a model consisting of virus-to-prokaryote ratio as explanatory and turbidity as conditional variable explained well FIC and VP determined from undiluted incubations (explained variation: 96%; S4 Table) but had no explanatory power for FIC and VP from virus dilution incubations. The virus-to-prokaryote ratio corrected for the influence of turbidity explained 62% of the variation in FIC and VP from undiluted incubations (S4 Table).

### Prokaryotic growth and turnover times of prokaryotes and viruses

PG in undiluted incubations was significantly higher compared to virus dilution incubations (Mann-Whitney test: *U* = 3.0, *p* = 0.0018; S5 Table). Nevertheless, prokaryotic mortality and PG corrected for prokaryotic mortality was similar in both approaches (Mann-Whitney test: prokaryotic mortality: *U* = 1.8, *p* = 0.0685; PG corrected for prokaryotic mortality: *U* = 1.2, *p* = 0.2387; S5 Table). Prokaryotic turnover time varied between 2.0–13.0 d in undiluted and between 3.6–15.6 d in virus dilution incubations (Table 2). Viral turnover time ranged from 2.0–11.8 d and 24.1–84.3 d in undiluted and virus dilution incubations, respectively (Table 2). Prokaryotic and viral turnover time were significantly lower in undiluted as compared to virus dilution incubations (prokaryotic turnover time: Mann-Whitney test: *U* = 3.1, *p* = 0.0014, *N* = 18; viral turnover time: Mann-Whitney test: *U* = 5.1, *p* < 0.0001, *N* = 18).

## Discussion

### Temporal variability in viral abundance–causes and consequences for rate estimates and FIC

In a typical one-step growth curve experiment with a specific prokaryotic taxon infected by a single virus taxon, one would expect an initial increase in viral abundance followed by a decrease in viral abundance, provided viral reinfection in the experiment is prevented by sufficiently low numbers of host cells and viruses. However, in all undiluted and virus dilution incubations in this study, viral abundance was much more variable over time (e.g., S1 Fig) and never followed the scenario just described. The cause for this contrast between our incubations and a single host-single virus taxon growth experiment is that we were studying a complex prokaryotic community, where a specific fraction of the prokaryotic cells was infected by an even more diverse virus community. Many of these host cells might have been in varying stages of viral infection (from attachment of the infecting virus to just before lysis of the host cell), some host cells might even have been infected by more than one virus taxon [25], and many viruses might have been different in latency period, burst size, and decay rates [18]. Thus, the temporal variation in bulk viral abundance in undiluted and virus dilution incubations in this study is a result of this enormous variation in the samples. Also, it has been shown that such variability in viral abundance in virus dilution incubations is not due to artifacts of the incubation, but is repeatable and has ecological relevance [23].

Acknowledging that viral abundance is temporally variable in such incubations has two important consequences. First, gathering data on a fine temporal scale (here we sampled every 5 h over a period of 40 h) should be preferable as the results will be more accurate. Second, more data create the problem that the results of linear least-squares regression analysis may not be supported by statistics anymore. As an example, when using the calculation method outlined in S1 Table for the specific case shown in S1 Fig, viral production yields 3.03×10^4^ viruses mL^-1^ h^-1^. Drawing a linear least-squares regression line through the same data puts viral production at 0.45×10^4^ viruses mL^-1^ h^-1^, an order of magnitude lower than our estimate. Additionally, the linear least-squares regression for the data in S1 Fig is not supported by statistics as the coefficient of determination *R*^*2*^ = 0.15 and *p* = 0.30 (i.e., 15% of the data are explained by the line that has a probability of 30% to have arisen accidentally). In all cases of virus dilution and undiluted incubations reported in this study, linear least-squares regression analyses yielded lower rate estimates compared to the calculation method illustrated in S1 Fig (data not shown) and in most cases these regression analyses were not supported by statistics (*R*^*2*^ < 0.50 and *p* > 0.05). Thus, we have adopted the "piece-wise" calculation method [23] for our data to estimate VP, VD, and FIC (S1 Table, S1 Fig). Given that the same arguments with respect to statistics also hold for data on the temporal development of prokaryotic abundance in our incubations and for the sake of comparability to rates obtained from variability in viral abundance, we also used the calculation method outlined in S1 Table and S1 Fig for estimating PG and prokaryotic mortality.

Viral abundance at any given time is a consequence of viral production and decay. Thus, increasing viral abundance in our incubations is a result of lower viral decay compared to viral production and decreasing viral abundance is due to lower viral production compared to viral decay. Consequently our data from undiluted and virus dilution incubations have to be interpreted as net estimates.

### Two approaches to estimate virus-induced mortality of prokaryotes with vastly different results

The most relevant finding of this study is that VP and FIC were not correlated between both approaches (see results section “Comparing data obtained from undiluted and virus dilution incubations”) and that VP and FIC were significantly higher in undiluted than in virus dilution incubations (Figs 3 and 4). High virus-host encounter rates due to high virus-to-prokaryote ratios allowed new virus infections to occur in undiluted incubations (S4 Table), resulting in significantly higher VP and FIC compared to the virus dilution incubations (Figs 3 and 4), with lower virus-host encounter rates due to reduced virus-to-prokaryote ratios. We also would like to point out that FLC could only be calculated once based on data from the undiluted approach, but never from virus dilution incubations. Nevertheless, this finding does not exclude the possibility of lysogenically infected prokaryotic cells in the Baltic Sea redoxcline as not all lysogenic viruses are inducible by mitomycin C [42]. Mitomycin C may cause substantial increases in size of the affected cells, because it blocks complete replication of the genome and consequently cell division ([43] and CW, personal observation). Given that new viral infections significantly influence VP and FIC in undiluted incubations (S4 Table), we believe that the sole estimate of FLC is the result of enhanced viral reinfection due to substantially larger prokaryotic host cells. Also, viral abundance at the beginning of the virus dilution incubations averaged 30% of *in situ* abundance, well within the dilution levels previously reported for similar incubations (e.g., [27,44,45]). In summary, we are confident that new viral infections during the time course of the virus dilution incubations were successfully prevented. Thus, the loss of viruses in virus dilution incubations was only compensated by viruses from infections prior to setting up the experiments; VP corrected for VD was significantly lower in virus dilution compared to undiluted incubations (S5 Table). Grazing as a source of mortality needs to be considered only for undiluted incubations in oxygenated waters [2], as water used for the virus dilution approach has been pre-filtered over 3 μm pore size filters, excluding most protistan grazers. Nevertheless, prokaryotic mortality was similar in both approaches (S5 Table). If grazing as a source of prokaryotic mortality had an influence in the undiluted incubations, prokaryotic mortality should decrease from the oxic to the anoxic zone. However, this trend was only observed at Landsort Deep 1, where prokaryotic mortality was substantially higher in the suboxic compared to the transition zone. No trend in prokaryotic mortality was found at Gotland Deep, and highest prokaryotic mortality was observed in the deepest anoxic zone at Landsort Deep 2 (S5 Table). Thus, in summary, patterns of prokaryotic mortality over depth in undiluted incubations as well as a comparison of prokaryotic mortality between both approaches are not compatible with appreciable protistan grazing [2] in undiluted incubations. Furthermore, sample manipulations necessary for virus dilution incubations might have changed overall prokaryotic growth compared to undiluted incubations. Indeed, PG in undiluted incubations was significantly higher and the resulting prokaryotic turnover time significantly lower compared to virus dilution incubations (S5 Table, Table 2), suggesting that high VP in undiluted incubations (Fig 3) might have stimulated prokaryotic growth by releasing organic carbon and other nutrients through cell lysis [46]. Additionally, the lower PG in virus dilution than in undiluted incubation indicates that filtration and concentration procedures in preparation of the virus dilution approach did not artificially alter prokaryotic growth by potentially leaching carbon from filters and plastic components. However, PG corrected for prokaryotic mortality, was similar in both approaches (S5 Table). The influence of different incubation volumes (20–1000 mL) on prokaryotic net growth (in the sense of PG corrected for prokaryotic mortality) was previously studied by Hammes and colleagues [47] in batch culture experiments for up to 5 days. These authors found no evidence that the volume of the incubations had any discernible effect on prokaryotic net growth. However, CARD-FISH data suggest that the relative abundance of *Bacteria* was substantially affected by the conditions in undiluted incubations but not in virus dilution incubations (S2 Fig).

In summary, we found significant differences in FIC, VP, VD, VP corrected for VD, PG, and the relative abundance of *Bacteria* between both approaches (S5 Table, Figs 3 and 4 and S2 Fig). The major advantage of the virus dilution approach is the isolation of the source of the studied viruses, allowing to estimate VP and FIC at the time of sampling without interference of other processes during the incubation period. In contrast, VP and FIC obtained from undiluted incubations are influenced by a number of uncontrollable processes during the incubation period, particularly of viral reinfection (S4 Table). Thus, it is unreasonable to compare VP and FIC obtained from undiluted incubations of different samples as not only VP and FIC per se may differ but also processes (e.g., nutrient recycling, particle attachment) indirectly affecting VP and FIC during the incubation period. This argument is further supported by the finding that VP and FIC within transition and anoxic zones differed among sampling sites when estimated by undiluted incubations in contrast to virus dilution incubations, where no differences were found (Figs 3 and 4). For these reasons, the complete lack of a correlation in VP and FIC between both approaches is comprehensible, if not expectable. In conclusion, we consider the virus dilution approach to be the more reliable approach to estimate VP and FIC. The undetectability of FLC based on data from the virus dilution approach suggests that lysogenic viruses do not contribute substantially to the virus community of the Baltic Sea redoxcline or if present were not inducible by mitomycin C (see also [2]). For the remainder of this discussion we refer to data from the virus dilution approach unless otherwise noted.

### Abundances, VP and FIC in comparison to previously published data from the same and other environments

Prokaryotic and viral abundances in the Baltic Sea were similar to previous studies in the same environment [2,19]. Also, prokaryotic abundances of surface waters of the North Sea [23], the North Adriatic Sea [48], and the Canadian Arctic Shelf [49] were comparable to the Baltic Sea (S6 Table). However, prokaryotic abundance of the Baltic Sea was much lower compared to the Chesapeake Bay [50] and was an order of magnitude higher than in the deep Atlantic Ocean [51]. In contrast, viral abundance in the Baltic Sea was lower than in the North Sea [23] and the Chesapeake Bay [50], comparable to the Canadian Arctic Shelf [49], and higher than in the North Adriatic Sea [48] and the deep Atlantic Ocean [51] (S6 Table). VP in virus dilution incubations (1.2–3.2×10^4^ mL^-1^ h^-1^; Fig 3) was similar to VP estimated from incubation-independent TEM observations by Weinbauer et al. (0.1–4.1×10^4^ mL^-1^ h^-1^) [19]. Furthermore, it was comparable to VP reported for the Canadian Shelf [49] and the Atlantic Ocean [51] but was lower than in the North Sea [23], the North Adriatic Sea [48], and the Northern Baltic Sea [27], and 2–3 orders of magnitude lower compared to the Chesapeake Bay [50] (S6 Table). However, FIC in virus dilution incubations was consistently low (≤11%; Fig 4), confirming earlier data from the same environment (FIC: 2–19%; [2]). Although FLC was detected by Weinbauer and colleagues (4–44%, [19]), it was not detected by Anderson and colleagues [2] and was only detected once in this study (47% in undiluted incubations in the transition zone at Landsort Deep 2). The discrepancies between our data (Figs 3 and 4) and Weinbauer and colleagues [19] might reflect differences in incubation and calculation of FIC and FLC. However, we cannot exclude the possibility that hydrological differences due to a major inflow event of North Sea water into the Baltic Sea in the year 2003 [3] were partly responsible for the observed differences in FIC and FLC reported by Weinbauer and colleagues (September 1998, [19]), the experiments of Anderson and colleagues (September 2009, [2]), and this study conducted in June 2012.

### High viral abundance as a result of exceptionally low VD_INT_

Low FIC (Fig 4) indicates that, despite high *in situ* viral abundance (1.2–1.9×10^7^ mL^-1^; Table 2), viruses are not a major mortality factor for prokaryotes, not even in the absence of protistan grazing [2]. Moreover, we could not detect virus decay in particle-free water (VD_INT_). Ultraviolet radiation as a source of virus decay can be excluded in the Baltic Sea redoxcline due to the high turbidity of the water column [52]; certainly so in our experiments as incubations were performed in the dark. Our inability to estimate VD_INT_ in ultra-filtered water suggests that only two other potential sources of virus decay were relevant in our experiments: prokaryotes secreting extracellular enzymes causing enzymatic viral decay and particles as potential viral attachment sites [9,53], both of which are effectively removed by ultra-filtration. Viral turnover time in virus dilution incubations (25.0–84.3 d) was up to ~26-times higher than in undiluted incubations (Table 2) and is among the highest estimates ever recorded for epipelagic waters (S6 Table). The data indicate that the Baltic Sea redoxcline constitutes an epipelagic environment where low virus production coincides with low viral decay, resulting in high viral abundance.

### Resource allocation strategies of prokaryotic viruses in the Baltic Sea redoxcline

The burst size depends on host characteristics [31,54] and has been shown experimentally to represent a virus strain-specific property [18]. Alternatively to transmission electron microscopy, we used changes in prokaryotic and viral abundance to empirically estimate burst size in our virus dilution incubations (S6 Table). Specifically, we assumed that VP and prokaryotic mortality were solely due to cell lysis caused by lytic viruses. Empirically-determined burst size ranged from 1–17 (average: 10; S6 Table), resulting in an increase of FIC by a factor of 2.8, on average. Viral turnover time (i.e., viral abundance divided by VP) does not depend on the burst size unlike FIC. Nevertheless, burst size and viral turnover time from previous studies [23,27,47,49,50,51] together with our data were negatively correlated (Spearman rank correlation coefficient: *r* = -0.83, *p* < 0.0001, *N* = 46; S6 Table). Even without other decay mechanisms, capsids of double-stranded DNA viruses experience stress due to the large amount of nucleic acids packaged in them, resulting in the build-up of pressure inside the capsids [17]. De Paepe and Taddei [18] have demonstrated that the burst size (i.e., the multiplication rate within the host) and the ability of viral capsids to withstand stress (i.e., survival of viruses in the environment determined by capsid thickness) are negatively related. These authors found that decay rates of viruses infecting prokaryotes are mainly determined by capsid thickness and the density of the packaged genome. In contrast to other environments (e.g., the North Sea; [23]), VD_INT_ of viruses from the Baltic Sea redoxcline was below the detection limit, suggesting that viruses found in the study area are either sturdily built or that the stress due to the packaged genome is small. In the study area high viral abundance in combination with low VP resulted in some of the highest viral turnover times reported for marine epipelagic environments (S6 Table). In combination with the negative relationship between viral turnover time and the low, empirically-determined burst size we conclude that prokaryotic viruses found in the Baltic Sea redoxcline resolve the trade-off in resource allocation by mostly investing in stress defense, i.e., presumably thick capsids but low burst size.

## Conclusions

VP and FIC estimated from undiluted incubations were much higher compared to data from virus dilution incubations (Figs 3 and 4), confirming our initial hypothesis. Most importantly, VP and FIC were not correlated between both approaches, indicating that uncontrollable processes (e.g., new viral infections during the experiment; S4 Table) in undiluted incubations dramatically affect the results. Based on virus dilution incubations, VP and FIC were low and stable throughout the redoxcline (Figs 3 and 4), resulting in exceptionally long viral turnover times (24–84 d, Table 2), coinciding with VD_INT_ below the detection limit and low empirically-determined burst size (1–17; S6 Table). Thus, the Baltic Sea redoxcline is the first identified epipelagic environment where high viral abundance is the result of low VP and low VD_INT_. Given a strong negative relationship between burst size and viral turnover times across a wide range of environments (S6 Table), the data are compatible with the notion that prokaryotic viruses of the Baltic Sea redoxcline invest the available limited resources mostly into stress defense (strong capsids, low VD_INT_, long viral turnover times) rather than into proliferation (low VP, low burst size). Given the apparent lack of a significant prokaryotic mortality factor in the Baltic Sea redoxcline, prokaryotic abundance may be controlled by nutrient limitation, especially so in the anoxic zone.

## Supporting information

S1 Fig Changes in prokaryotic and viral abundance during an incubation experiment, conducted with seawater from the anoxic zone at Gotland Deep using the virus dilution approach. The Figure shows local minima and maxima of prokaryotic (P_minn_ and P_maxn_) and viral abundance (V_minn_ and V_maxn_) and their respective time points (TP_minn_, TP_maxn_, TV_minn_, and TV_maxn_; see also S1 Table).
(PDF)

S2 Fig Relative abundances of *Bacteria* (A,C,E) and *Crenarchaeota* (B,D,F) in *in situ* samples (dark grey bars) and at termination of experimental incubations performed at Gotland Deep (A,B), Landsort Deep 1 (C,D), and Landsort Deep 2 (E,F). Undiluted (white bars) and virus dilution incubations (grey bars) were performed with water from the oxic (OZ), suboxic (SZ), transition (TZ), and anoxic zone (AZ). Data for experimental incubations are given as the average of duplicate incubations and error bars represent the range.
(PDF)

S1 File Prokaryotic and viral abundances for undiluted and virus dilution experiments. Abundances were determined every 5 h after start of the incubations (t_0_).
(XLS)

S1 Table Equations to calculate prokaryotic growth (PG), prokaryotic mortality, PG corrected for prokaryotic mortality, virus production (VP), viral decay (VD), VP corrected for VD, and FIC. Parameters were calculated from temporal changes in prokaryotic and viral abundance during the incubations where P_maxn_ and P_minn_ correspond to the n^th^ local maximum and minimum, respectively, in prokaryotic abundance, V_maxn_ and V_minn_ to the nth local maximum and minimum, respectively, in viral abundance, TP_maxn_ and TP_minn_ to the time point of the nth local maximum and minimum, respectively, in prokaryotic abundance, and TV_maxn_ and TV_minn_ to the time point of the nth local maximum and minimum, respectively, in viral abundance. Equations correspond to S1 Fig and were adapted to other patterns of prokaryotic and viral abundance if necessary. PG, prokaryotic mortality, PG corrected for prokaryotic mortality, VP, VD, and VP corrected for VD were corrected for the difference between *in situ* and initial prokaryotic abundance when estimated from the virus dilution incubation.
(PDF)

S2 Table Kruskal-Wallis test for differences in the relative abundance of bacteria and crenarchaeota. The table gives the test statistic (*H*) and the corresponding *p*-value of a Kruskal-Wallis test performed with the relative abundance of *Bacteria* and *Crenarchaeota* of *in situ* samples and data from undiluted and virus dilution incubations at the end of the incubation period. Results were assumed to be statistically significant as *p* ≤ 0.05.
(PDF)

S3 Table Variation partitioning of FIC and VP based on nutrient concentrations and water masses. The table gives the fraction (%) of the variation of the frequency of infected cells (FIC) and virus production (VP) explained by the specific model and its corresponding *p*-value (n.a.: not applicable). The concentrations of NO_2_, NH_4_, and PO_4_ served as explanatory variables, collectively referred to as nutrients. The matrix of conditional variables consisted of temperature and salinity, representative of the sampled water mass. Results are considered significant at *p* ≤ 0.05.
(PDF)

S4 Table Variation partitioning of FIC and VP based on virus-to-prokaryote ratio and turbidity. The table gives the fraction (%) of the variation of the frequency of infected cells (FIC) and virus production (VP) explained by the specific model and its corresponding *p*-value (n.a.: not applicable). The virus-to-prokaryote ratio served as explanatory and turbidity as conditional variable. Results are considered significant at *p* ≤ 0.05.
(PDF)

S5 Table Rates of prokaryotic growth (PG), prokaryotic mortality, PG corrected for prokaryotic mortality, viral decay (VD), and virus production corrected for VD (VP corrected for VD). The table gives average (*Avg*) and range of prokaryotic growth (PG; N×10^3^ mL^-1^ h^-1^), prokaryotic mortality (N×10^3^ mL^-1^ h^-1^), prokaryotic growth corrected for prokaryotic mortality (PG corrected for prokaryotic mortality; N×10^3^ mL^-1^ h^-1^), viral decay (VD; N×10^4^ mL^-1^ h^-1^), and virus production corrected for VD (VP corrected for VD; N×10^4^ mL^-1^ h^-1^) estimated from duplicates of undiluted and virus dilution incubations, respectively, at Gotland Deep, Landsort Deep 1, and Landsort Deep 2.
(PDF)

S6 Table Compilation of prokaryotic and viral abundance, virus-to-prokaryote ratio, virus production, viral turnover time, viral turnover, and burst size. The tables give prokaryotic abundance (PA, N×10^5^ mL^-1^), viral abundance (VA, N×10^6^ mL^-1^), the virus-to-prokaryote ratio (VPR), virus production (VP, N×10^4^ mL^-1^ h^-1^), viral turnover time (VTT, d), viral turnover (VT, d^-1^), and burst size (BS) from previously published studies and this study using the virus dilution approach (VDA). If BS was not obtained by the authors, the applied burst size and its source is given. BS was either obtained by transmission electron microscopy (TEM) or calculated from incubation experiments (empirical) as the ratio of VP to prokaryotic mortality or VP to lysed active prokaryotic cells (NuCC).^a^ If specific parameters were not reported directly, they were calculated from provided data as follows: VPR = VA / PA, VTT = VA / VP, VT = 1 / VTT.
(PDF)
