# Supplementary material for: High viral abundance as a consequence of low viral decay in the Baltic Sea redoxcline
Source: PLoS One. 2017 Jun 8;12(6):e0178467. doi: 10.1371/journal.pone.0178467 (PMC5464540; doi:10.1371/journal.pone.0178467)
Supplement: S1 Fig — The Figure shows local minima and maxima of prokaryotic (Pminn and Pmaxn) and viral abundance (Vminn and Vmaxn) and their respective time points (TPminn, TPmaxn, TVminn, and TVmaxn; see also S1 Table). (PDF) [file pone.0178467.s001.pdf]

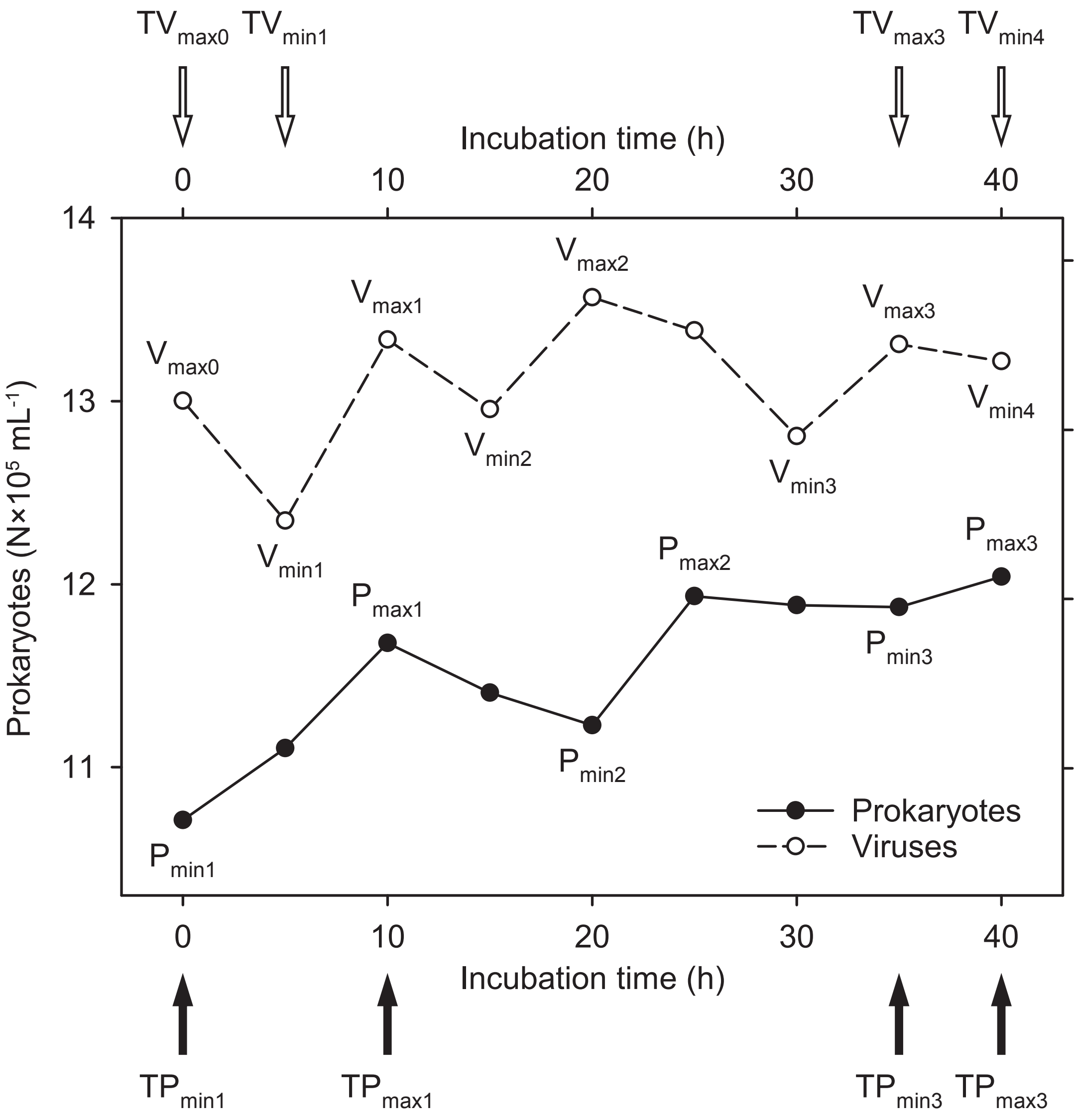

Fig S1. Changes in prokaryotic and viral abundance during an incubation experiment, conducted with seawater from the anoxic zone at Gotland Deep using the virus dilution approach. The Figure shows local minima and maxima of prokaryotic (P_{minn} and P_{maxn}) and viral abundance (V_{minn} and V_{maxn}) and their respective time points (TP_{minn}, TP_{maxn}, TV_{minn}, and TV_{maxn}; see also Table S1).
