# Supplementary material for: High viral abundance as a consequence of low viral decay in the Baltic Sea redoxcline
Source: PLoS One. 2017 Jun 8;12(6):e0178467. doi: 10.1371/journal.pone.0178467 (PMC5464540; doi:10.1371/journal.pone.0178467)
Supplement: S2 Fig — Undiluted (white bars) and virus dilution incubations (grey bars) were performed with water from the oxic (OZ), suboxic (SZ), transition (TZ), and anoxic zone (AZ). Data for experimental incubations are given as the average of duplicate incubations and error bars represent the range. (PDF) [file pone.0178467.s002.pdf]

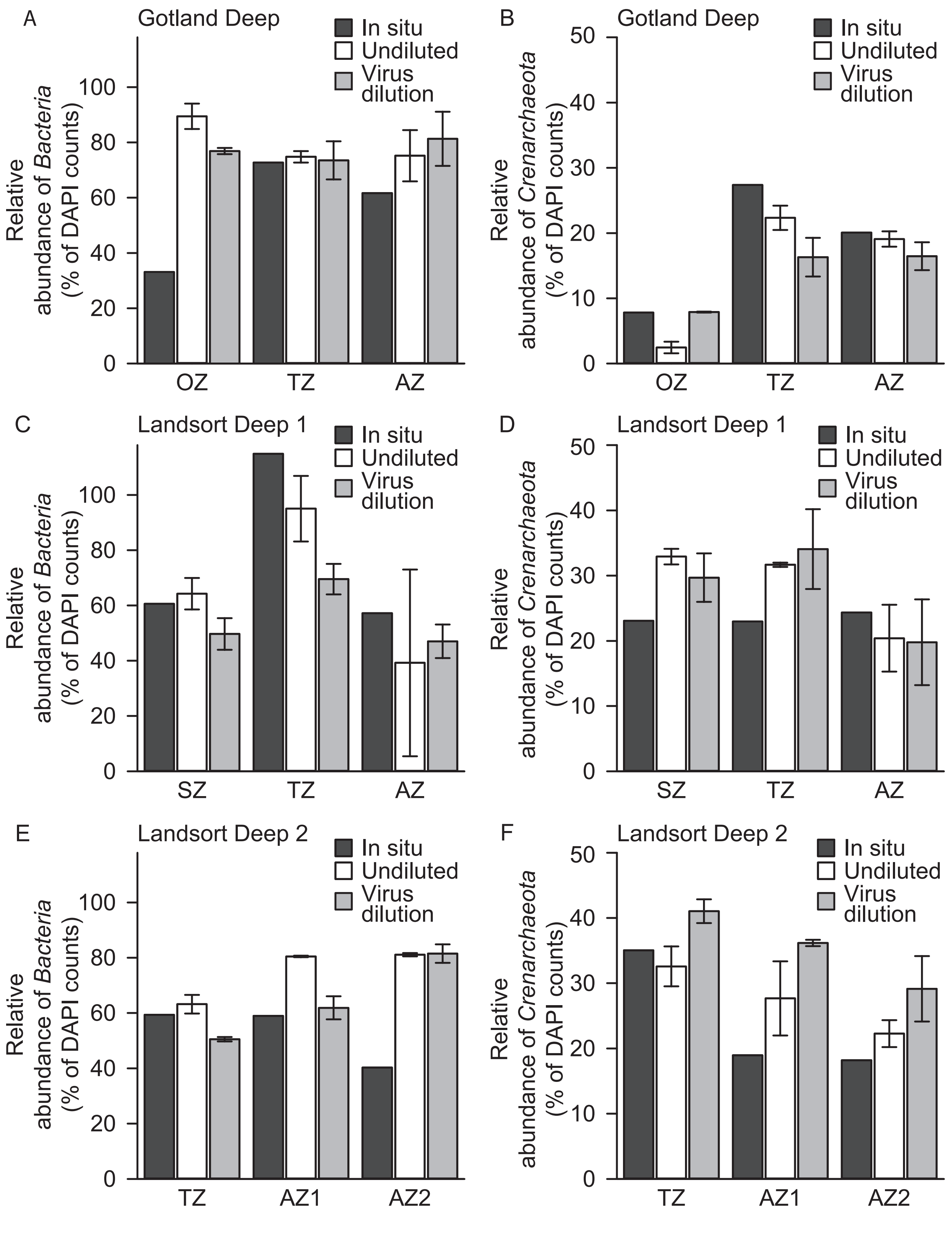

Fig S2. Relative abundances of *Bacteria* (A,C,E) and *Crenarchaeota* (B,D,F) in in situ samples (dark grey bars) and at termination of experimental incubations performed at Gotland Deep (A,B), Landsort Deep 1 (C,D), and Landsort Deep 2 (E,F). Undiluted (white bars) and virus dilution incubations (grey bars) were performed with water from the oxic (OZ), suboxic (SZ), transition (TZ), and anoxic zone (AZ). Data for experimental incubations are given as the average of duplicate incubations and error bars represent the range.
