# Supplementary material for: High viral abundance as a consequence of low viral decay in the Baltic Sea redoxcline
Source: PLoS One. 2017 Jun 8;12(6):e0178467. doi: 10.1371/journal.pone.0178467 (PMC5464540; doi:10.1371/journal.pone.0178467)
Supplement: S1 Table — Parameters were calculated from temporal changes in prokaryotic and viral abundance during the incubations where Pmaxn and Pminn correspond to the nth local maximum and minimum, respectively, in prokaryotic abundance, Vmaxn and Vminn to the nth local maximum and minimum, respectively, in viral abundance, TPmaxn and TPminn to the time point of the nth local maximum and minimum, respectively, in prokaryotic abundance, and TVmaxn and TVminn to the time point of the nth local maximum and minimum, respectively, in viral abundance. Equations correspond to S1 Fig and were adapted to other patterns of prokaryotic and viral abundance if necessary. PG, prokaryotic mortality, PG corrected for prokaryotic mortality, VP, VD, and VP corrected for VD were corrected for the difference between in situ and initial prokaryotic abundance when estimated from the virus dilution incubation. (PDF) [file pone.0178467.s004.pdf]

Table S1. Equations to calculate prokaryotic growth (PG), prokaryotic mortality, PG corrected for prokaryotic mortality, virus production (VP), viral decay (VD), VP corrected for VD, and FIC.

Equation no.	Equation	Unit
1	PG = $[(P_{max1} – P_{min1}) + (P_{max2} – P_{min2}) + (P_{max3} – P_{min3})] / (TP_{max3} – TP_{min1})$	cells mL^{-1} h^{-1}
2 *	prokaryotic mortality = $[(P_{min2} – P_{max1}) + (P_{min3} – P_{max2})] / (TP_{min3} – TP_{max1})$	cells mL^{-1} h^{-1}
3	PG corrected for prokaryotic mortality = PG + prokaryotic mortality	cells mL^{-1} h^{-1}
4	prokaryotic turnover time = $(P_{in\ situ}$ / PG) / 24 h	d
5	VP = $[(V_{max1} – V_{min1}) + (V_{max2} – V_{min2}) + (V_{max3} – V_{min3})] / (TV_{max3} – TV_{min1})$	viruses mL^{-1} h^{-1}
6 *	VD = $[(V_{min1} – V_{max0}) + (V_{min2} – V_{max1}) + (V_{min3} – V_{max2}) + (V_{min4} – V_{max3})] / (TV_{min4} – TV_{max0})$	viruses mL^{-1} h^{-1}
7	VP corrected for VD = VP + VD	viruses mL^{-1} h^{-1}
8	viral turnover time = $(V_{in\ situ}$ / VP) / 24h	d
9	FIC = 100 × $[(V_{max1} – V_{min1}) + (V_{max2} – V_{min2}) + (V_{max3} – V_{min3})]$ / (burst size × P_0)	% of in situ abundance

Parameters were calculated from temporal changes in prokaryotic and viral abundance during the incubations where P_{maxn} and P_{minn} correspond to the nth local maximum and minimum, respectively, in prokaryotic abundance, V_{maxn} and V_{minn} to the nth local maximum and minimum, respectively, in viral abundance, TP_{maxn} and TP_{minn} to the time point of the nth local maximum and minimum, respectively, in prokaryotic abundance, and TV_{maxn} and TV_{minn} to the time point of the nth local maximum and minimum, respectively, in viral abundance. Equations correspond to Fig. S1 and were adapted to other patterns of prokaryotic and viral abundance if necessary. PG, prokaryotic mortality, PG corrected for prokaryotic mortality, VP, VD, and VP corrected for VD were corrected for the difference between in situ and initial prokaryotic abundance when estimated from the virus dilution incubation.

* Please note that calculation of these rates results in negative values.
