# Supplementary material for: High viral abundance as a consequence of low viral decay in the Baltic Sea redoxcline
Source: PLoS One. 2017 Jun 8;12(6):e0178467. doi: 10.1371/journal.pone.0178467 (PMC5464540; doi:10.1371/journal.pone.0178467)
Supplement: S2 Table — The table gives the test statistic (H) and the corresponding p-value of a Kruskal-Wallis test performed with the relative abundance of Bacteria and Crenarchaeota of in situ samples and data from undiluted and virus dilution incubations at the end of the incubation period. Results were assumed to be statistically significant as p ≤ 0.05. (PDF) [file pone.0178467.s005.pdf]

Table S2. Kruskal-Wallis test for differences in the relative abundance of *Bacteria* and *Crenarchaeota*

Depth zone	Relative abundance of *Bacteria*			Relative abundance of *Crenarchaeota*		
	H	*p*	*N*	*H*	*p*	*N*
Oxic Zone	3.6	0.1653	5	3.0	0.2231	5
Suboxic Zone	3.0	0.2231	5	2.4	0.3012	5
Transition Zone	2.0	0.3633	15	0.4	0.8395	15
Anoxic Zone	4.3	0.1173	20	0.7	0.7097	20
All zones	6.9	0.0315	45	1.1	0.5685	45

The table gives the test statistic (H) and the corresponding p-value of a Kruskal-Wallis test performed with the relative abundance of *Bacteria* and *Crenarchaeota* of in situ samples and data from undiluted and virus dilution incubations at the end of the incubation period. Results were assumed to be statistically significant as $p \leq 0.05$.
