# Supplementary material for: High viral abundance as a consequence of low viral decay in the Baltic Sea redoxcline
Source: PLoS One. 2017 Jun 8;12(6):e0178467. doi: 10.1371/journal.pone.0178467 (PMC5464540; doi:10.1371/journal.pone.0178467)
Supplement: S3 Table — The table gives the fraction (%) of the variation of the frequency of infected cells (FIC) and virus production (VP) explained by the specific model and its corresponding p-value (n.a.: not applicable). The concentrations of NO2, NH4, and PO4 served as explanatory variables, collectively referred to as nutrients. The matrix of conditional variables consisted of temperature and salinity, representative of the sampled water mass. Results are considered significant at p ≤ 0.05. (PDF) [file pone.0178467.s006.pdf]

Table S3. Variation partitioning of FIC and VP based on nutrient concentrations and water masses

Parameters	Undiluted FIC and VP		Virus dilution FIC and VP	
	Fraction	*p*	Fraction	*p*
Nutrients and water mass	81	0.2536	93	0.0455
Nutrients not corrected for water mass	62	0.1644	75	0.0598
Water mass not corrected for nutrients	62	0.0759	52	0.1294
Nutrients	19	0.5209	41	0.0313
Water mass correlated nutrients	43	n.a.	34	n.a.
Water mass	19	0.3489	18	0.1521
Unexplained	19	n.a.	7	n.a.

The table gives the fraction (%) of the variation of the frequency of infected cells (FIC) and virus production (VP) explained by the specific model and its corresponding *p*-value (n.a.: not applicable). The concentrations of NO_2, NH_4, and PO_4 served as explanatory variables, collectively referred to as nutrients. The matrix of conditional variables consisted of temperature and salinity, representative of the sampled water mass. Results are considered significant at $p \leq 0.05$.
