# Supplementary material for: High viral abundance as a consequence of low viral decay in the Baltic Sea redoxcline
Source: PLoS One. 2017 Jun 8;12(6):e0178467. doi: 10.1371/journal.pone.0178467 (PMC5464540; doi:10.1371/journal.pone.0178467)
Supplement: S4 Table — The table gives the fraction (%) of the variation of the frequency of infected cells (FIC) and virus production (VP) explained by the specific model and its corresponding p-value (n.a.: not applicable). The virus-to-prokaryote ratio served as explanatory and turbidity as conditional variable. Results are considered significant at p ≤ 0.05. (PDF) [file pone.0178467.s007.pdf]

Table S4. Variation partitioning of FIC and VP based on virus-to-prokaryote ratio and turbidity

Parameters	Undiluted FIC and VP		Virus dilution FIC and VP	
	Fraction	*p*	Fraction	*p*
Virus-to-prokaryote ratio and turbidity	96	0.0005	39	0.4837
Virus-to-prokaryote ratio not corrected for turbidity	92	0.0014	38	0.2436
Turbidity not corrected for virus-to-prokaryote ratio	34	0.0758	6	0.6097
Virus-to-prokaryote ratio	62	0.0002	33	0.3265
Turbidity correlated virus-to-prokaryote ratio	30	n.a.	5	n.a.
Turbidity	4	0.0531	1	0.8771
Unexplained	4	n.a.	61	n.a.

The table gives the fraction (%) of the variation of the frequency of infected cells (FIC) and virus production (VP) explained by the specific model and its corresponding *p*-value (n.a.: not applicable). The virus-to-prokaryote ratio served as explanatory and turbidity as conditional variable. Results are considered significant at $p \leq 0.05$.
