# Supplementary material for: High viral abundance as a consequence of low viral decay in the Baltic Sea redoxcline
Source: PLoS One. 2017 Jun 8;12(6):e0178467. doi: 10.1371/journal.pone.0178467 (PMC5464540; doi:10.1371/journal.pone.0178467)
Supplement: S5 Table — The table gives average (Avg) and range of prokaryotic growth (PG; N×103 mL-1 h-1), prokaryotic mortality (N×103 mL-1 h-1), prokaryotic growth corrected for prokaryotic mortality (PG corrected for prokaryotic mortality; N×103 mL-1 h-1), viral decay (VD; N×104 mL-1 h-1), and virus production corrected for VD (VP corrected for VD; N×104 mL-1 h-1) estimated from duplicates of undiluted and virus dilution incubations, respectively, at Gotland Deep, Landsort Deep 1, and Landsort Deep 2. (PDF) [file pone.0178467.s008.pdf]

Table S5. Rates of prokaryotic growth (PG), prokaryotic mortality, PG corrected for prokaryotic mortality, viral decay (VD), and virus production corrected for VD (VP corrected for VD)

Sampling locations	Depth zone	PG				Prokaryotic mortality				PG corrected for prokaryotic mortality				VD				VP corrected for VD			
		Undiluted		Virus dilution		Undiluted		Virus dilution		Undiluted		Virus dilution		Undiluted		Virus dilution		Undiluted		Virus dilution	
		Avg	Range	*Avg*	Range	*Avg*	Range	*Avg*	Range	*Avg*	Range	*Avg*	Range	*Avg*	Range	*Avg*	Range	*Avg*	Range	*Avg*	Range
Gotland Deep	Oxic Zone	4.9	1.4	2.6	0.4	-3.6	1.9	-1.3	0.1	1.3	0.5	1.4	0.4	-15.0	3.9	-2.6	0.5	-2.3	1.7	-0.5	0.1
	Transition Zone	6.7	0.9	5.2	0.9	-2.4	0.8	-3.4	0.5	4.3	0.0	1.9	0.4	-12.1	0.6	-2.2	0.1	3.1	2.4	-0.8	0.2
	Anoxic Zone	7.3	1.4	4.3	1.2	-2.6	1.5	-2.8	1.5	4.6	0.1	1.5	0.3	-4.1	0.5	-1.4	0.0	1.5	1.1	0.0	0.7
Landsort Deep 1	Suboxic Zone	6.4	1.8	2.2	0.6	-7.3	0.0	-1.2	0.8	-0.8	1.8	0.9	0.2	-15.1	6.2	-6.1	1.2	2.9	3.6	-4.9	2.1
	Transition Zone	6.8	1.4	4.0	0.2	-6.2	0.9	-2.3	0.2	0.6	0.5	1.7	0.0	-35.6	4.4	-7.3	1.6	-0.7	3.1	-4.2	3.2
	Anoxic Zone	8.7	0.5	6.1	0.1	-4.8	2.9	-2.9	0.2	3.8	2.4	3.3	0.1	-11.8	0.6	-3.1	0.8	7.3	0.0	-0.2	0.1
Landsort Deep 2	Transition Zone	5.5	1.7	2.2	0.4	-5.7	2.7	-2.3	0.5	-0.3	1.0	-0.1	0.9	-10.4	0.0	-6.3	0.1	10.3	1.8	-3.1	1.9
	Anoxic Zone1	3.4	1.4	2.8	1.0	-2.0	1.3	-2.0	0.5	1.3	0.0	0.8	0.5	-11.6	2.3	-5.1	0.3	-1.7	2.7	-2.7	1.1
	Anoxic Zone 2	20.4	1.0	6.9	2.7	-12.5	0.3	-14.0	3.3	8.0	1.3	-7.1	6.1	-9.6	3.2	-3.3	1.4	2.2	2.3	-1.3	2.3

The table gives average (*Avg*) and range of prokaryotic growth (PG; N×10^3 mL^{-1} h^{-1}), prokaryotic mortality (N×10^3 mL^{-1} h^{-1}), prokaryotic growth corrected for prokaryotic mortality (PG corrected for prokaryotic mortality; N×10^3 mL^{-1} h^{-1}), viral decay (VD; N×10^4 mL^{-1} h^{-1}), and virus production corrected for VD (VP corrected for VD; N×10^4 mL^{-1} h^{-1}) estimated from duplicates of undiluted and virus dilution incubations, respectively, at Gotland Deep, Landsort Deep 1, and Landsort Deep 2. Please note that rates of decline are given as negative values.
