# Supplementary material for: High viral abundance as a consequence of low viral decay in the Baltic Sea redoxcline
Source: PLoS One. 2017 Jun 8;12(6):e0178467. doi: 10.1371/journal.pone.0178467 (PMC5464540; doi:10.1371/journal.pone.0178467)
Supplement: S6 Table — The tables give prokaryotic abundance (PA, N×105 mL-1), viral abundance (VA, N×106 mL-1), the virus-to-prokaryote ratio (VPR), virus production (VP, N×104 mL-1 h-1), viral turnover time (VTT, d), viral turnover (VT, d-1), and burst size (BS) from previously published studies and this study using the virus dilution approach (VDA). If BS was not obtained by the authors, the applied burst size and its source is given. BS was either obtained by transmission electron microscopy (TEM) or calculated from incubation experiments (empirical) as the ratio of VP to prokaryotic mortality or VP to lysed active prokaryotic cells (NuCC).a If specific parameters were not reported directly, they were calculated from provided data as follows: VPR = VA / PA, VTT = VA / VP, VT = 1 / VTT. (PDF) [file pone.0178467.s009.pdf]

Table S6 part 1. Compilation of prokaryotic and viral abundance, virus-to-prokaryote ratio, virus production, viral turnover time, viral turnover, and burst size

Environment	PA	VA	VPR	VP	VTT	VT	Method	Source	BS	Determination of BS
North Sea, western site	12.0	21.0	18	20.0	4.38[a]	0.23[a]	VDA	Winter et al., 2004	30	TEM
North Sea, southern site	15.0	94.0	63	51.0	7.68[a]	0.13[a]	VDA	Winter et al., 2004	21	TEM
North Sea, northern site	6.0	23.0	38	3.0	31.94[a]	0.03[a]	VDA	Winter et al., 2004	22	TEM
North Adriatic Sea, station A118, surface	18.8	9.5	5	486.6	0.08	12.24[a]	VDA	Bongiorni et al., 2005	109	empirical (VP/NuCC)
North Adriatic Sea, station D051, surface	13.9	11.2	8	127.2	0.37	2.73[a]	VDA	Bongiorni et al., 2005	105	empirical (VP/NuCC)
North Adriatic Sea, station SS08, surface	3.4	3.2	9	115.4	0.11	8.76[a]	VDA	Bongiorni et al., 2005	41	empirical (VP/NuCC)
Baltic Sea, station RA3	20.0	22.5	11[a]	12.5	7.50[a]	0.13[a]	VDA	Holmfeldt et al., 2010	28	TEM (Parada et al., 2006)
Baltic Sea, station A5	13.0	12.0	9[a]	45.8	1.09[a]	0.92[a]	VDA	Holmfeldt et al., 2010	28	TEM (Parada et al., 2006)
Baltic Sea, station A13	14.0	15.0	11[a]	25.0	2.50[a]	0.40[a]	VDA	Holmfeldt et al., 2010	28	TEM (Parada et al., 2006)
Baltic Sea, station B3	24.0	19.0	8[a]	10.4	7.61[a]	0.13[a]	VDA	Holmfeldt et al., 2010	28	TEM (Parada et al., 2006)
Baltic Sea, station C3	12.0	12.0	10[a]	2.1	23.81[a]	0.04[a]	VDA	Holmfeldt et al., 2010	28	TEM (Parada et al., 2006)
Baltic Sea, station C14	22.0	14.5	7[a]	8.3	7.28[a]	0.14[a]	VDA	Holmfeldt et al., 2010	28	TEM (Parada et al., 2006)
Chesapeake Bay, February	26.0	110.0	45	710.0	0.71[a]	1.40	VDA	Winget et al., 2011	130	empirical (VP/prok. mortality)
Chesapeake Bay, March	29.0	97.0	42	1200.0	0.91[a]	1.10	VDA	Winget et al., 2011	150	empirical (VP/prok. mortality)
Chesapeake Bay, April	33.0	110.0	41	770.0	0.50[a]	2.00	VDA	Winget et al., 2011	120	empirical (VP/prok. mortality)
Chesapeake Bay, May	42.0	110.0	31	250.0	2.08[a]	0.48	VDA	Winget et al., 2011	14	empirical (VP/prok. mortality)
Chesapeake Bay, June	96.0	200.0	21	830.0	0.91[a]	1.10	VDA	Winget et al., 2011	130	empirical (VP/prok. mortality)
Chesapeake Bay, July	110.0	170.0	19	890.0	0.77[a]	1.30	VDA	Winget et al., 2011	18	empirical (VP/prok. mortality)
Chesapeake Bay, August	92.0	190.0	19	820.0	0.77[a]	1.30	VDA	Winget et al., 2011	61	empirical (VP/prok. mortality)
Chesapeake Bay, September	69.0	80.0	12	310.0	1.10[a]	0.91	VDA	Winget et al., 2011	23	empirical (VP/prok. mortality)
Chesapeake Bay, October	55.0	200.0	37	460.0	1.22[a]	0.82	VDA	Winget et al., 2011	48	empirical (VP/prok. mortality)
Canadian Arctic Shelf, station 912	15.0	27.0	18[a]	32.0	2.00[a]	0.50	VDA	Payet et al., 2013	18	TEM (Middelboe et al. 2002)
Canadian Arctic Shelf, station 803	14.0	24.0	17[a]	25.0	4.00[a]	0.25	VDA	Payet et al., 2013	18	TEM (Middelboe et al. 2002)
Canadian Arctic Shelf, station 718	7.0	7.0	10[a]	8.0	3.33[a]	0.30	VDA	Payet et al., 2013	18	TEM (Middelboe et al. 2002)
Canadian Arctic Shelf, station 650	4.0	7.0	18[a]	3.0	10.00[a]	0.10	VDA	Payet et al., 2013	18	TEM (Middelboe et al. 2002)
Canadian Arctic Shelf, station 415	7.5	8.0	11[a]	6.0	20.00[a]	0.05	VDA	Payet et al., 2013	18	TEM (Middelboe et al. 2002)
Canadian Arctic Shelf, station 200	6.0	9.0	15[a]	5.0	10.00[a]	0.10	VDA	Payet et al., 2013	18	TEM (Middelboe et al. 2002)
Canadian Arctic Shelf, station 106	5.5	15.0	27[a]	4.0	20.00[a]	0.05	VDA	Payet et al., 2013	18	TEM (Middelboe et al. 2002)

Table S6 part 2. Compilation of prokaryotic and viral abundance, virus-to-prokaryote ratio, virus production, viral turnover time, viral turnover, and burst size

Environment	PA	VA	VPR	VP	VTT	VT	Method	Source	BS	Determination of BS
Atlantic Ocean, northern section, OMZ	0.6	1.0	17	4.2	0.95[a]	1.05[a]	VDA	Muck et al., 2014	30	TEM (Parada et al., 2006)
Atlantic Ocean, northern section, LNADW	0.1	0.4	38	1.2	1.42[a]	0.70[a]	VDA	Muck et al., 2014	30	TEM (Parada et al., 2006)
Atlantic Ocean, northern section, AABW	0.1	0.5	36	1.4	1.42[a]	0.70[a]	VDA	Muck et al., 2014	30	TEM (Parada et al., 2006)
Atlantic Ocean, Vema Fracture Zone, OMZ	0.8	1.3	16	4.1	1.31[a]	0.76[a]	VDA	Muck et al., 2014	30	TEM (Parada et al., 2006)
Atlantic Ocean, Vema Fracture Zone, LNADW	0.1	0.4	35	1.3	1.40[a]	0.71[a]	VDA	Muck et al., 2014	30	TEM (Parada et al., 2006)
Atlantic Ocean, Vema Fracture Zone, AABW	0.2	0.6	38	1.4	1.73[a]	0.58[a]	VDA	Muck et al., 2014	30	TEM (Parada et al., 2006)
Atlantic Ocean, eastern section, OMZ	0.8	1.0	13	4.0	1.10[a]	0.91[a]	VDA	Muck et al., 2014	30	TEM (Parada et al., 2006)
Atlantic Ocean, eastern section, LNADW	0.1	0.5	39	0.9	2.08[a]	0.48[a]	VDA	Muck et al., 2014	30	TEM (Parada et al., 2006)
Atlantic Ocean, eastern section, AABW	0.1	0.5	39	1.1	1.94[a]	0.51[a]	VDA	Muck et al., 2014	30	TEM (Parada et al., 2006)
Baltic Sea, Gotland Deep, oxic zone	2.1	19.2	90	2.1	42.33	0.02	VDA	this study	17	empirical (VP/prok. mortality)
Baltic Sea, Gotland Deep, transition zone	9.0	16.6	18	1.4	53.56	0.02	VDA	this study	4	empirical (VP/prok. mortality)
Baltic Sea, Gotland Deep, anoxic zone	7.3	14.7	20	1.3	69.83	0.01	VDA	this study	8	empirical (VP/prok. mortality)
Baltic Sea, Landsort Deep 1, suboxic zone	5.4	13.8	25	1.2	84.33	0.01	VDA	this study	14	empirical (VP/prok. mortality)
Baltic Sea, Landsort Deep 1, transition zone	9.8	15.6	16	3.2	27.94	0.04	VDA	this study	13	empirical (VP/prok. mortality)
Baltic Sea, Landsort Deep 1, anoxic zone	8.9	15.8	18	2.9	24.08	0.04	VDA	this study	11	empirical (VP/prok. mortality)
Baltic Sea, Landsort Deep 2, transition zone	5.4	11.7	22	3.2	25.03	0.04	VDA	this study	13	empirical (VP/prok. mortality)
Baltic Sea, Landsort Deep 2, anoxic zone 1	8.8	14.3	16	2.4	28.42	0.04	VDA	this study	12	empirical (VP/prok. mortality)
Baltic Sea, Landsort Deep 2, anoxic zone 2	9.3	15.2	16	2.1	37.22	0.03	VDA	this study	2	empirical (VP/prok. mortality)

The tables give prokaryotic abundance (PA, $N\times10^5$ mL^{-1}), viral abundance (VA, $N\times10^6$ mL^{-1}), the virus-to-prokaryote ratio (VPR), virus production (VP, $N\times10^4$ mL^{-1} h^{-1}), viral turnover time (VTT, d), viral turnover (VT, d^{-1}), and burst size (BS) from previously published studies and this study using the virus dilution approach (VDA). If BS was not obtained by the authors, the applied burst size and its source is given. BS was either obtained by transmission electron microscopy (TEM) or calculated from incubation experiments (empirical) as the ratio of VP to prokaryotic mortality or VP to lysed active prokaryotic cells (NuCC).

[a] If specific parameters were not reported directly, they were calculated from provided data as follows: VPR = VA / PA, VTT = VA / VP, VT = 1 / VTT.
